# Complicated Infective Endocarditis Limited to a Chiari Network

**DOI:** 10.1155/2018/3837825

**Published:** 2018-06-26

**Authors:** Olufolahan Carrena, Oluchukwu Oluoha, Amr Wahba, Derek Eshun, Maria Endsley, Henry Okafor

**Affiliations:** ^1^Meharry Medical College, Nashville, TN, USA; ^2^Vanderbilt University Medical Center, Nashville, TN, USA

## Abstract

**Introduction:**

The Chiari network is an uncommon vestigial structure of the heart that is often clinically insignificant. We present an unusual case of infective endocarditis affecting only the Chiari network in a patient who presented with septic emboli to the lungs and brain.

**Case summary:**

A 61-year-old man was admitted with a 2-month history of hemoptysis, pleuritic chest pain, and right upper extremity numbness and weakness. He was found to have multifocal bilateral pulmonary opacities and an abscess collection in the brain. Blood cultures grew *Streptococcus intermedius* and transthoracic echocardiogram (TTE) was normal. Subsequent transesophageal echocardiogram (TEE) revealed an 8.3 × 4.6 mm vegetation arising from the Chiari network, close to the right atrial appendage, without involvement of the tricuspid valve or any of the other valves. There were no atrial or ventricular septal defects. He was treated with appropriate antibiotics with improvement of symptoms. Repeat imaging showed improvement of the lung opacities, but not the brain abscess, warranting transfer to another hospital for neurosurgical intervention.

**Conclusion:**

The diagnosis and management of isolated Chiari network endocarditis require a high index of clinical suspicion. A multidisciplinary approach incorporating both medical and surgical approaches where necessary is essential for optimal outcome.

## 1. Case Presentation

A 61-year-old male patient presented to the emergency room (ER) on account of a 3-month history of hemoptysis as well as a 2-week history of right upper extremity weakness and numbness.

Hemoptysis was associated with pleuritic right-sided chest pain as well as orthopnea and had been previously treated with two courses of empiric antibiotics and steroids at an outside hospital without improvement prompting his presentation to our emergency room.

Weakness and numbness of the right upper extremity were initially associated with a painful right palmar rash which was resolved with self-administered topical corticosteroids at home.

Past medical history was significant for untreated latent tuberculosis (TB) diagnosed about forty years prior to this presentation as well as coronary artery disease requiring stent placement twice in the past.

On presentation, the patient was not in acute distress but was tachycardic at 100 beats per minute, with a respiratory rate of 16 cycles per minute and an oxygen saturation of 100 percent on room air. Blood pressure was 144/87 millimeters of mercury, and temperature was 99 degrees Fahrenheit.

On physical examination, he was afebrile and had normal breath sounds and heart sounds without murmurs. Neurological examination was notable for reduced sensation to light but not crude touch over the right hand with reduced strength of 4/5 in that in same extremity. Laboratory studies were significant for a white blood cell count of 18,400 per microliter with 76.3 percent neutrophils, an erythrocyte sedimentation rate of 90 millimeters per hour, and a C-reactive protein level of 167 milligrams per liter.

**Figure 1:**
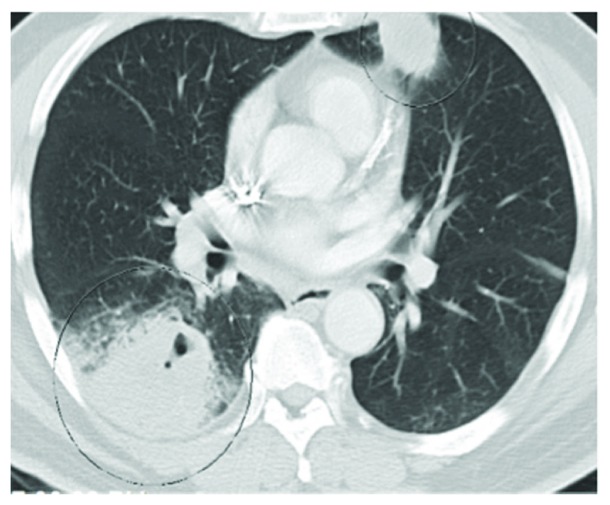
Septic pulmonary emboli.

Imaging done on admission revealed multifocal lung opacities (Figure 1) concerning for community-acquired multifocal pneumonia (possibly secondary to a resistant organism as patient had completed courses of cefdinir and levofloxacin at the outside hospital without resolution of his symptoms). Other considerations were for possible septic emboli of undetermined source as well as reactivated pulmonary tuberculosis given his long-standing history of latent TB with multiple positive skin purified protein derivative tests in the past.

**Figure 2:**
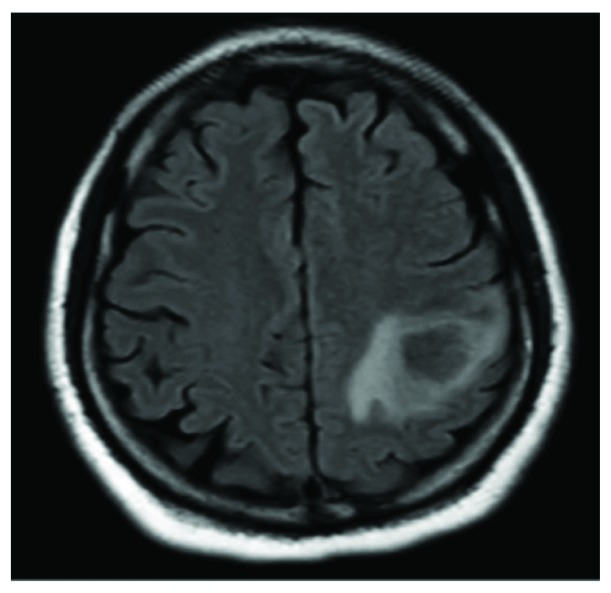
Septic focus in the brain.

**Figure 3:**
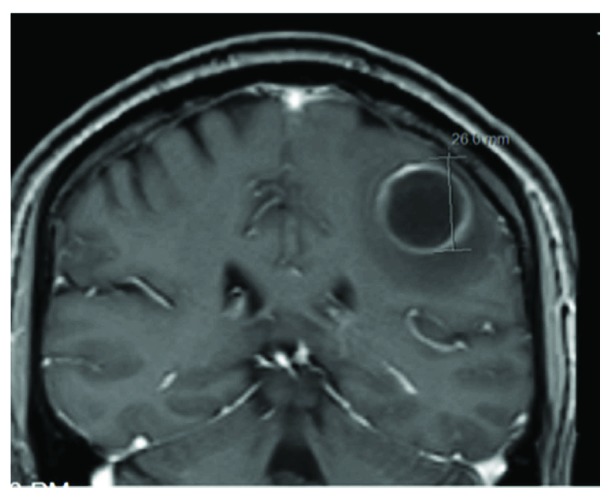
Parietal lobe lesion.

CT scan of the head without contrast was concerning for a possible infarct of unclear age, and a follow-up MRI revealed possible abscess collection (Figures 2 and 3).

He was initially placed on airborne isolation with empiric first-line antituberculosis agents (which were discontinued after 6 negative acid-fast bacilli sputum samples were obtained) as well as empiric antibiotics (vancomycin and piperacillin/tazobactam) for possible multifocal pneumonia, based on recommendations of the infectious disease consultants. Two blood cultures, obtained on admission, returned positive and grew *Streptococcus intermedius* sensitive to ceftriaxone, levofloxacin, tetracycline, erythromycin, clindamycin, and vancomycin. A transthoracic echocardiogram showed normal left ventricular systolic function and normal valves without regurgitations, stenosis or, vegetations.

**Figure 4:**
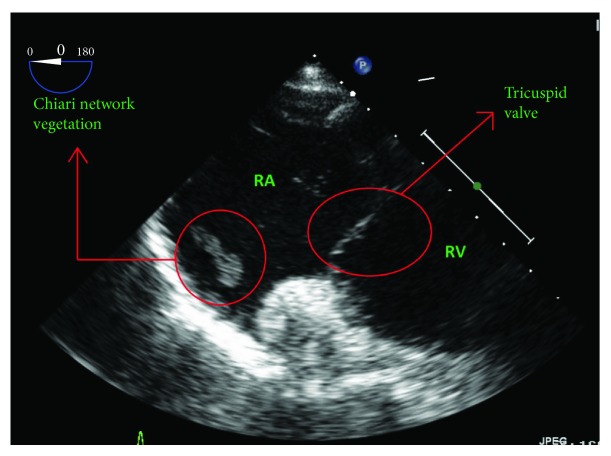
Transesophageal echocardiogram image showing the Chiari network vegetation separated from the tricuspid valve.

A subsequent transesophageal echocardiogram (TEE) revealed an 8.3 × 4.6 mm oscillating dumbbell-shaped vegetation arising from the Chiari network, close to the os of the right atrial appendage (Figure 4), without evidence of valvular vegetations, insufficiency, or a patent foramen ovale.

A diagnosis of infective endocarditis was made based on the modified Duke criteria with two separate blood cultures positive for *Streptococcus intermedius*, echocardiographic findings of an oscillating intracardiac mass, consistent with a vegetation, and septic pulmonary and intracranial lesions [1]. Medical management was chosen at the time of the diagnosis of this Chiari network endocarditis with septic emboli to the lungs and brain, with plans for repeat imaging with a transesophageal echo after six weeks of appropriate antibiotic therapy.

Antibiotic coverage was thus narrowed and tailored to the culprit organism with ceftriaxone, and gentamycin was added for endocarditis synergy per infectious disease team recommendations. Clinical and laboratory evidences of improvement including resolution of the hemoptysis and right upper extremity weakness as well as reduction in the white blood cell count from 18,400 per microliter on admission to 6400 per microliter and in the erythrocyte sedimentation rate from 90 millimeters per hour to 28 millimeters per hour as well as the C reactive protein from 167 milligrams per liter to 0.9 milligrams per liter were documented.

Interval reduction in the size of the lung lesions but not the brain lesions was noted after four weeks of parenteral antibiotic treatment, and neurosurgical evaluation for possible evacuation of the abscess was thus recommended. The patient was transferred to an outside hospital where those services were readily available. He had an image-guided stereotactic drainage of the abscess via a left parietal burr hole, with initial gram stain of the fluid showing gram-positive cocci, but the culture had no growth after 5 days probably because the patient had been on appropriate antibiotics for four weeks prior to the drainage procedure.

The patient completed six weeks of antibiotic therapy at the outside hospital and was discharged home in a stable clinical state but did not follow up as recommended.

## 2. Discussion

The Chiari network is a mobile, fenestrated anatomic variant seen in the right atrium of about 1.5–3% of the population [2, 3] and has been described as an embryonic remnant of the right valve of the venous sinus [4].

Even though it is usually of no significance and discovered incidentally during transesophageal echocardiography for other indications or at autopsy, historical associations with some conditions such as a patent foramen ovale, with more intense right-to-left shunting than controls, atrial septal aneurysm and recurrent arterial embolic events with a cardiac source have been described in the literature [2, 3]. Infective endocarditis affecting the Chiari network is a rare finding which has been seldom described in the literature [5], and isolated involvement of the Chiari network, while sparing the other heart valves, is clearly a much rarer entity.

Our patient presented with features of the complications of infective endocarditis with an initial negative TTE which was followed by a TEE, done on account of a high index of suspicion, which eventually revealed the vegetations attached solely to the Chiari network, sparing the valves on both the right and left sides of the heart. This is in contrast to many previously reported cases in which other heart valves were notably involved [5, 6], sometimes on both sides of the heart.

Complications of infective endocarditis such as the multilobar lung abscesses and single brain abscess, noted in our patient, which likely occurred through hematogenous spread from the primary focus of infection on the Chiari malformation have been described in the past as well. This spread is thought to be facilitated in patients with atrial septal defects or other right-to left shunts [5] which was not demonstrated on the echocardiograms of this patient. The lack of a physically demonstrable shunt however does not preclude hematogenous spread as demonstrated in our index case.

The right upper extremity palmar rash reported by the patient on admission, which was resolved with topical corticosteroids prior to presentation, was concerning for possible Osler's nodes or Janeway lesions which are expected skin findings of a painful papular and painless macular rash, respectively, in patients with infective endocarditis. It is however difficult to make definitive comments on this as it was not present on admission. It is also unlikely for Osler's nodes or Janeway lesions to resolve with topical corticosteroids without adequate treatment tailored to the causative agent of the infective endocarditis, as described in a similar case, where the skin manifestations we noted to have resolved within 8–10 weeks of therapy (a 12-week course of antibiotics had been planned for the patient in that case report on account of methicillin-resistant *Staphylococcus* being the causative organism) [5]. Our patient's blood cultures grew *Streptococcus intermedius*, sensitive to ceftriaxone, for which at least 6 weeks of antibiotic therapy was the recommended treatment approach.

The decision for the medical versus surgical approach to the treatment of Chiari network endocarditis has no set guidelines in terms of size or extent of the vegetation; however, Payne et al. in 2003 described a surgical approach to a patient who already had another indication for cardiac surgery (three vessel disease requiring coronary artery bypass grafting) [3]. Complete resolution with medical therapy alone has also been described in a patient with Chiari network endocarditis also affecting the aortic valve [5], even though the dimensions of the vegetation on the Chiari network in that report were much larger, (28 × 8 mm) compared to those of our patient (8.3 × 4.6 mm).

A medical approach was thus prudent, with plans to reevaluate with interval imaging of the abscesses and also a repeat TEE upon completion of the 6-week course of recommended antibiotics. This is consistent with the 2015 American Heart Association (AHA) 2015 Scientific Statement on infective endocarditis management in adults which stated that repeat echocardiography at the time of antimicrobial therapy completion was a class IIa recommendation with evidence level C (meaning that it was based partly on divergent expert opinion, and additional studies with focused objectives were needed to support this practice) [1].

Our patient's case is unique in the fact that he presented with features of the complications, which were later tied together by the definitive diagnosis. Medical management of the Chiari network endocarditis with antibiotics resulted in clinical improvement, reduction in the size of the lung lesions, and resolution of the right upper extremity weakness. The persistent brain abscesses required neurosurgical evacuation which the patient received at an outside hospital.

## 3. Conclusion

In future cases with infective endocarditis affecting the Chiari network, either alone or in conjunction with other heart valves, a medical approach to treatment can be explored, while monitoring with appropriate imaging modalities, for the resolution of the vegetation itself and also that of any sites of complications from septic emboli. Complications from septic emboli, as seen in our patient, may warrant widening of the multidisciplinary approach to the care of these patients to involve the possibility of interventions to mitigate these complications.
